# Obesity induces morphological and functional changes in female reproductive system through increases in NF-κB and MAPK signaling in mice

**DOI:** 10.1186/s12958-021-00833-x

**Published:** 2021-09-24

**Authors:** Xiuxiu Gao, Yangyang Li, Zhuoyao Ma, Jia Jing, Zhengqing Zhang, Yue Liu, Zhide Ding

**Affiliations:** 1 Department of Histology, Embryology, Genetics and Developmental Biology, Shanghai Key Laboratory for Reproductive Medicine, Shanghai Jiao Tong University School of Medicine, Shanghai, 200025 China; 2 Department of Medical Laboratory Science, Shanghai Ninth People’s Hospital, Shanghai Jiao Tong University School of Medicine, Shanghai, 200011 China

**Keywords:** Obesity, High fat diet (HFD), Female fertility, Pro-inflammatory cytokines, Signal pathway

## Abstract

**Background:**

Recently, human infertility incidence is increasing in obese women causing it to become an emerging global health challenge requiring improved treatment. There is extensive evidence that obesity caused female reproductive dysfunction is accompanied by an endocrinological influence. Besides, systemic and tissue-specific chronic inflammatory status are common characteristics of obesity. However, the underlying molecular mechanism is unclear linking obesity to infertility or subfertility.

**Methods:**

To deal with this question, we created an obese mouse model through providing a high fat diet (HFD) and determined the fertility of the obese mice. The morphological alterations were evaluated in both the reproductive glands and tracts, such as uterus, ovary and oviduct. Furthermore, to explore the underlying mechanism of these functional changes, the expressions of pro-inflammatory cytokines as well as the activations of MAPK signaling and NF-κB signaling were detected in these reproductive tissues.

**Results:**

The obese females were successful construction and displayed subfertility. They accumulated lipid droplets and developed morphological alterations in each of their reproductive organs including uterus, ovary and oviduct. These pathological changes accompanied increases in pro-inflammatory cytokine expression levels of interleukin-6 (IL-6) and tumor necrosis factor-α (TNF-α) in all of these sites. Such effects also accompanied increases in nuclear factor kappa B (NF-kB) expression and mitogen-activated protein kinase (MAPK) signaling pathway stimulation based on uniform time dependent increases in the NF-κB (p-NF-κB), JNK (p-JNK), ERK1/2 (p-ERK) and p38 (p-p38) phosphorylation status.

**Conclusions:**

These HFD-induced increases in pro-inflammatory cytokine expression levels and NF-κB and MAPKs signaling pathway activation in reproductive organs support the notion that increases of adipocytes resident and inflammatory status are symptomatic of female fertility impairment in obese mice.

**Supplementary Information:**

The online version contains supplementary material available at 10.1186/s12958-021-00833-x.

## Background

The gradual rises in overweight and obesity prevalence has reached global epidemic proportions and is becoming a huge burden on the health care systems in many communities. Their prevalence rose 27.5% in adults and 47.1% in children respectively during the last three decades according to a study describing Global Burden of Disease in 2013 [1].

Obesity is associated with many deleterious and chronic diseases such as cardiovascular disease, diabetes, stroke, cancers, etc. There is extensive data showing that the frequency of ovulation and spontaneous conception decreases in obese women [2–5]. Other studies reported that obesity can alter the uterine morphology and fluid content, which leads to declines in implantation and fertilization [6, 7]. Besides, obesity is also associated with adverse obstetric outcomes, such as gestational diabetes, premature labor, preeclampsia and stillbirth [8]. On the other hand, experimental data from mouse models suggest that obesity can suppress oocyte maturation, increase both apoptosis of granulosa cells and oocyte dysfunction [9].

Obesity is defined as an abnormality resulting from increases in fat accumulation caused by maladaptive changes in lipid metabolism. In general, most research regarding lipid profile changes has focused on adipose tissue and adipocytes. Adipocytes have the primary function of controlling energy balance by storing lipids during periods of excessive food intake or depleting their stores during famine. Besides this function, adipocytes also play pivotal roles as an endocrine organ by secreting numerous factors including cytokines, chemokines and adipokines [10]. These different secretions have profound effects on physiological and pathological processes such as glucose metabolism, inflammatory responses, regulation of blood pressure and reproduction [11].

The reproductive events, including ovulation, menstruation, implantation and parturition, are commonly orchestrated by specific receptor-linked signaling pathways controlling expression of inflammatory cytokines, chemokines and lipid mediators [4, 12, 13]. Appropriate control of these signaling pathways is vital for sustaining normal functions, whereas their dysregulation can cause pathophysiological conditions and diseases to develop. Such changes can alter regulation of normal reproductive function which may disrupt the menstrual cycle [14] as well as induce endometriosis-associated infertility [15], intrauterine growth restriction [16], preeclampsia [16], recurrent miscarriage [17] and premature delivery [18]. There is evidence that obesity induced changes can be the result of disordered insulin signaling in the ovary and pituitary gland metabolic activity [19, 20]. Other studies suggest that disrupted normal reproductive behavior may instead stem from abnormal adipogenesis [21] and mitochondrial dysfunction in the ovarian follicular cells [22–24]. Recently, it has become apparent throughout the world that a chronic low-grade inflammatory status accompanies obesity in both humans and experimental animal models [13, 25, 26]. However, the underlying molecular mechanism in the obesity-induced low fertility animal model requires clarification even though some reports describe that there is a relationship between inflammatory status and female obesity [27, 28].

We show here that numerous morphological alterations develop in both the reproductive glands and tracts of high-fat diet (HFD) fed obese mice in comparison to their normal control. Furthermore, these pathological changes in the HFD mice accompanied subfertility due to impaired reproductive function. Underlying these functional changes, obesity led to a chronic inflammatory status due to sustained MAPK signaling and NF-κB activation associated with pro-inflammatory cytokine upregulation. Therefore, our study illustrates a previously unappreciated mechanism by which obesity impairs female fertility throughout the reproductive glands and tracts, and provide novel options for treating obesity caused female infertility.

## Material and methods

### Animals and obese model establishment

All animal experiments were approved by the Ethics Committee of Shanghai Jiao Tong University School of Medicine (NO. A2015–034 and NO. A2019–029) and performed in accordance with the International Guiding Principles for Biomedical Research Involving Animals, as promulgated by the Society for the Study of Reproduction. C57BL/6 female mice (3 weeks old) were purchased from Shanghai Laboratory Animal Center and then acclimated in a 12 h:12 h light: dark cycle under standard conditions (25 ± 2 °C and 50 ± 10% humidity) at least for 1 week prior to experimentation in the Animal Center of Jiao Tong University Medical School. Females were divided into two groups and were fed ad libitum. Thirty mice were fed a HFD in which 60% of its caloric value is derived from fat (Research Diets, New Brunswick, USA). Another Thirty mice were fed a normal diet (ND) in which fat provides only 10% of its caloric value. The protein, carbohydrate and fat constituents of both HFD and ND are provided in Table 1. The detailed ingredients of both feeds are provided in supplementary Table 1. Both groups were continuously fed for 10 weeks and the body weight of each mouse in both groups was recorded weekly.

**Table 1:** The formula of hight-fat diet and normal diet

	Normal diet (Product #D12450J)		High fat diet (Product #D12492)	
	gm%	Kcal%	gm%	Kcal%
**Protein**	19.20%	20	26.2	20
**Carbohydrate**	67.30%	70	26.3	20
**Fat**	4.30%	10	34.9	60
**Total (kcal/gm)**	3.85%	100	5.24	100

### Female mice fertility evaluation

After fed the HFD or ND for 10 weeks, 10 mice from each group were cohabitated with fertile males (10 weeks old) for 10 consecutive days, and they were then separated from one another. Every day during cohabitation, females were examined for vaginal plugs as evidence of mating. About 21 days later, the number of pups per litter delivered by each female was recorded and calculated as previous reported [29].

### Blood sample and subcutaneous fat tissue collections, and serum lipid analysis

Mice at the 14-week-old were euthanized with CO_2_ and then blood samples from each group were taken and kept at 4 °C for 2 h to evaluate clotting times. All the samples were centrifuged (15 min, 4 °C, 3000 g) and the serum was immediately stored at − 20 °C for future analysis. Subsequently, their abdominal cavities were exposed and the fat tissues were collected and weighed.

The serum lipid parameters including cholesterol (CHOL), triglycerides (TGL), high density lipoprotein (HDL), and low density lipoprotein (LDL) were measured with a Roche COBAS c 311 auto biochemistry analyzer (Roche Diagnostics, Mannheim, Germany) according to the instructions provided by the manufacturer.

### Histopathology and immunohistochemistry (IHC) analysis

For morphological analysis, reproductive tissues and liver specimens were fixed in 4% paraformaldehyde for 24 h and then embedded in paraffin. The paraffin-fixed samples were sliced into 5 μm thick sections and dewaxed and rehydrated according to standard procedures. Finally, the slides were stained with hematoxylin and eosin (H&E).

For evaluating the accumulation of fat droplets, freshly sliced sections of mice reproductive tissues were incubated in 60% isopropanol for 3 min, followed by incubation with Oil red O reagent for 10 min. The slices were washed with 60% isopropanol and water, respectively. Then they were stained with hematoxylin and mounted on glass slides.

IHC staining was employed to assess the expression levels of interleukin-6 (IL-6) and tumor necrosis factor (TNF-α) in the female genital system. Briefly, the paraffin sections of mice reproductive tissues were deparaffinized, rehydrated, unmasked and then incubated overnight at 4 °C with mouse monoclonal anti-IL-6 (1:200, Cell Signaling Technology, Beverley, MA, USA) and mouse monoclonal anti-TNF-α antibodies (1:200, Cell Signaling Technology). Subsequently, the sections were incubated with biotinylated anti-mouse secondary antibody (1:5000, Abgent, San Diego, CA, USA). Finally, all the slides including H&E stained sections, Oil red O stained sections and immunohistochemical stained sections were observed and images were captured under a light microscope (Olympus BX53; Olympus, Tokyo, Japan).

### Western blot analysis

Immunoblotting was performed as described previously [30]. Mice uteri, ovaries and oviducts were homogenized in RIPA lysis buffer (Thermo Fisher Scientific, Rockford, IL, USA) containing protease inhibitor cocktail (Roche Mannheim, Germany) on ice for 30 min followed by centrifugation at 12,000 g, for 10 min, at 4 °C. The proteins in the supernatant were collected and their concentrations were determined by the BCA Protein Assay Kit (Thermo Fisher Scientific). Protein samples (30 μg) were separated by 12.5% SDS-PAGE and then electrotransferred to polyvinylidene fluoride membranes (Millipore, Billerica, MA, Germany) using a semi-dry transfer apparatus (Bio-Rad, Hercules, CA, USA). Membranes were blocked with 5% skim milk for 1 h and then were incubated overnight at 4 °C with the following primary antibodies: JNK (SAPK/JNK, 1:1000, Cell Signaling Technology), p-JNK (phosphor-SAPK/JNK Thr183/Tyr185, 1:1000, Cell Signaling Technology), ERK1/2 (p44/42, 1:1000, Cell Signaling Technology), p-ERK (phosphor-p42/44 Thr202/Tyr204, 1:1000, Cell Signaling Technology), p38 (1:1000, Cell Signaling Technology), p-p38 (phospho-p38 Thr180/Tyr182, 1:1000, Cell Signaling Technology), NF-κB (p65, 1:800, Cell Signaling Technology), p-NF-κB (phospho-p65 Ser536, 1:600, Cell Signaling Technology), IL-6 (1:500, Cell Signaling Technology) and TNF-α (1:500, Cell Signaling Technology), and followed by 1 h incubation with the appropriate secondary antibodies conjugated to HRP (1:5000, Abgent, San Diego, CA, USA). Signals were generated by enhanced chemiluminescence (Millipore) and detected by luminescent image analyzer (GE imagination LAS 4000, GE Healthcare Bio-Sciences AB, Uppsala, Sweden). Meanwhile, β-actin (1:1000, Cell Signaling Technology) was included to validate protein loading equivalence.

### Quantitative real-time PCR analysis

Total RNA was extracted from the uterus, ovary and oviduct by using Trizol Reagent (Invitrogen, CA, USA), according to the manufacturer’s protocol. The primers of IL-6, TNF-α and β-actin were listed in Table 2, according to previous reported [25]. The complimentary DNA was prepared from a 10 μL reaction system with 500 ng RNA by using PrimeScript RT Master Mix reagent kit (Takara, Kyoto, Japan). SYBR Premix Ex Taq II (Takara) was applied to measure the IL-6 and TNF-α gene expression levels in the uterus, ovary and oviduct tissues by an ABI 7500 (Applied Biosystems, Foster City, CA, USA), while β-actin expression was used for gene expression normalization. PCR conditions were set as follows: 5 min at 95 °C, followed by 40 cycles at 95 °C for 15 s, 60 °C for 34 s. Finally, the data were analyzed by using the 2^−ΔΔCT^ method to measure the relative gene expression levels.

**Table 2:** PCR primer sequences

Name	Sequence	5′ – 3′
IL-6		
Forward primer	GGCGGATCGGATGTTGTGAT	
Reverse primer	GGACCCCAGACAATCGGTTG	
TNF-α		
Forward primer	CAGGCGGTGCCTATGTCTC	
Reverse primer	CGATCACCCCGAAGTTCAGTAG	
β-actin		
Forward primer	GTGACGTTGACATCCGTAAAGA	
Reverse primer	GCCGGACTCATCGTACTC	

### Statistical analysis

All statistical analyses were performed using the Statistical Package for the Social Sciences (SPSS) 20.0 (IBM, Armonk, NY), and the results are expressed as mean ± standard deviation (SD). The statistical difference between two groups was assessed using Student’s t-test. A two-sided t-test with *P <* 0.05 was used to establish significance between differences.

## Results

### Profiles of female obese mice model

**Fig. 1:**
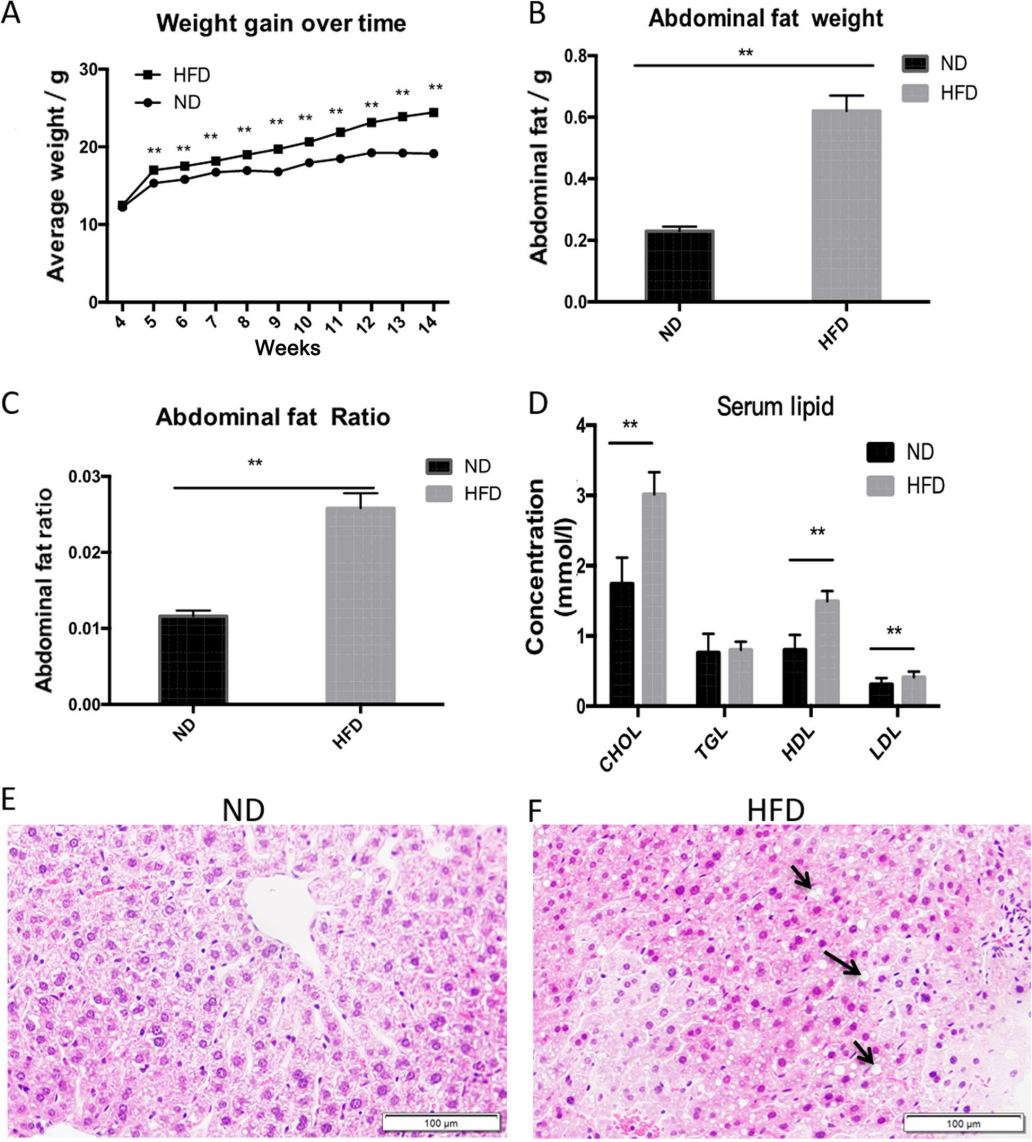
Metabolic and structural profile development in HFD fed mice. **A** Body weight gain of mice on HFD exceeded their counterpart fed instead a ND (*n* = 30, **P < 0.01). Abdominal fat weight (**B**), the ratio of abdominal fat weight to body weight (**C**) and serum lipids including CHOL, HDL and LDL in HFD mice content were also significantly higher than those in the ND control group. TGL levels in the HFD and ND groups were not different from one another (**D**). The data were present as mean ± SD (*n* = 20, **P < 0.01). **E**-**F** Hematoxylin and eosin (H&E) stained hepatic sections revealed a serious hepatic steatosis and many fat vacuoles in the HFD mice (**F**, arrows indicating fat vacuoles), but no morphological changes developed in liver sections of ND mice (**E**). Each group was consistently composed of three females. Scale bar = 100 μm

Female C57BL/6 mice continuously consumed either a HFD or a ND for 10 weeks. At the 5th week, the body weight difference between these two groups became significantly different, which persisted for the subsequent 9 weeks (Fig. 1A). In the 14th week, the HFD group weight was 24.44 ± 2.74 g whereas in the ND group it was 19.14 ± 1.28 g (*n* = 30, *P* < 0.01, Fig. 1A). Meanwhile, the abdominal fat weights and the ratio of fat mass to body weight were evaluated in both groups. The results showed significant increases of both parameters in HFD group (abdominal fat: 0.77 ± 0.19 g vs. 0.31 ± 0.15 g, *n* = 30, *P* < 0.01; the ratio of abdominal fat mass to body weight: 3.2 ± 0.7% vs. 1.4 ± 0.5%, n = 30, *P* < 0.01; Fig. 1B, C).

Additionally, increased serum lipid levels are signatures of obesity. Higher levels of CHOL, HDL and LDL were detected in the HFD group than those in the ND fed mice (CHOL: 3.04 ± 0.36 vs. 2.16 ± 0.69 mmol/L; HDL: 1.52 ± 0.17 vs. 1.03 ± 0.37 mmol/L; LDL: 0.44 ± 0.10 vs. 0.34 ± 0.09 mmol/L, *n* = 20, *P* < 0.01). However, the TGL levels were not different between the two groups (0.80 ± 0.08 vs. 0.77 ± 0.24 mmol/L, n = 20, *P* = 0.57; Fig. 1D).

Mammalian liver is the primary organ of fat metabolism and accumulation. Morphological analysis of H&E staining indicated pronounced hepatic steatosis and many fat vacuoles inclusions in the HFD fed mice (*n* = 3, Fig. 1E and F). Such changes are indicative of abnormal lipid metabolism development in the HFD fed group.

### Obesity effects on female fertility

**Fig. 2:**
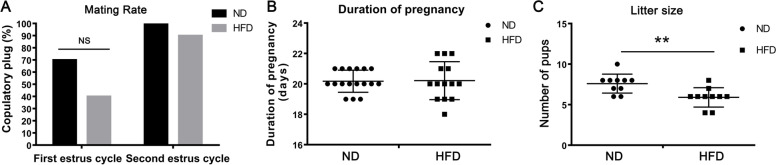
Obesity impairs female fertility. **A** The mating rates of ten females in each group during their two different estrus cycles. Data were presented as percentage and statistically analyzed by χ2 analysis (n = 20, *P* = 0.18 and 0.30 respectively). **B** Comparison of the pregnancy duration between the HFD females and ND females. The data were presented as mean ± SD (n = 20, *P* = 0.92). **C** The litter sizes of the females in their two breeding trials revealed that the average number of pups per litter was smaller in mice on a HFD than those fed a ND. This difference is associated declines in fertility and structural alterations in the female obese reproductive system. The results were reported as mean ± SD (*n* = 17 in ND group and *n* = 13 in HFD group, **P < 0.01)

To assess the effects of consuming a HFD on female fertility, breeding trials were performed twice on 10 female mice through natural mating with fertile males. The females were checked for the presence of vaginal plugs as evidence of mating. In their first estrus cycles to perform breeding trials, the mating rate of the HFD group (4/10) was a slight decrease, but not significant, in comparison to ND group (7/10, *P* = 0.18) (Fig. 2A). Meanwhile, in the second breeding trials, the mating rates of two groups were still similar (10/10 vs. 9/10, *P* = 0.30) (Fig. 2A). Moreover, the pregnancy duration of the females was also calculated and the results showed no significant difference between two groups (ND, 20.18 ± 0.17 days, *n* = 17; HFD, 20.21 ± 0.33 days; *n* = 13; Fig. 2B). Specially, the number of pups per litter was significant less in HFD group (5.69 ± 1.18, n = 13) than that in ND groups (8.35 ± 1.41, n = 17, *P* < 0.01; Fig. 2C). The reduced litter size on a HFD suggested that obesity is detrimental to normal function of the reproductive system, which contributed to a decline in fertility in this group.

### Obesity effects on the lipid accumulation in female reproductive organs

**Fig. 3:**
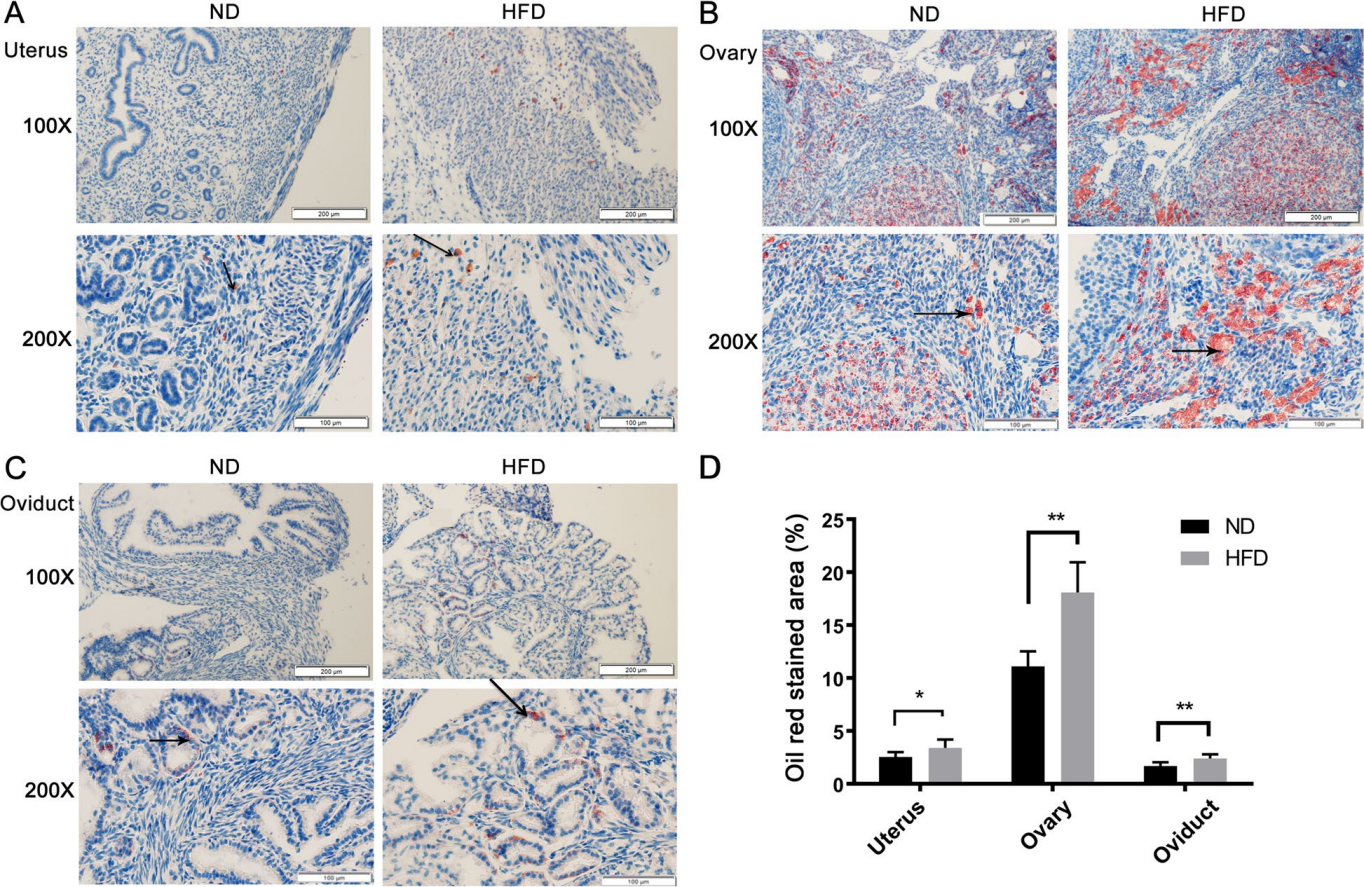
Lipid accumulation in female reproductive organs of obese mice. **A**-**C** Oil red O stains lipids in frozen histological slides. Differences in such staining indicated that the HFD fed mice uterus (**A**), ovary (**B**) and oviduct (**C**) contained much more lipids than their counterpart fed a ND. Data were evaluated from six independent experiments. Scale bar = 100 μm, 200 μm, respectively (arrows indicating lipids accumulation). **D** Quantitative analysis of Oil red O staining area (%) in slides. The results were reported as mean ± SD (*n* = 6, * *P* < 0.05, **P < 0.01)

Morphological analyses of the Oil Red O dye staining patterns showed that massive amounts of lipids accumulated in uterine, ovarian and oviduct tissues in HFD fed mice (Fig. 3A-C, arrows indicated the Oil Red O staining), which was significantly greater than that in their counterpart on a ND (Fig. 3D). Such alterations in these structures were indicative of extensive pathological development.

### Obesity effects on the morphology of female reproductive organs

**Fig. 4:**
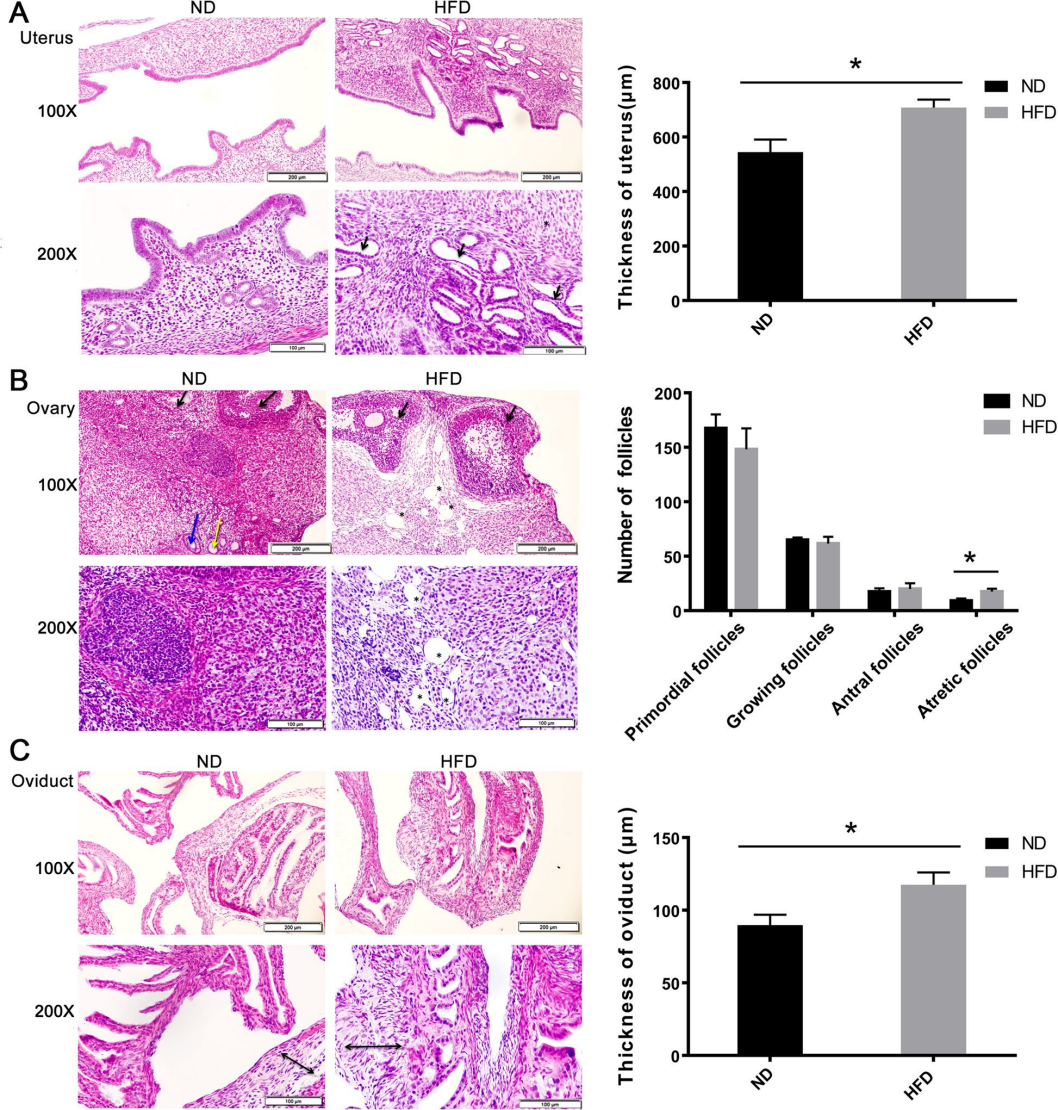
Morphological changes in obese reproductive organs. **A** H&E stained uterine sections of HFD and ND fed mice. Uterine stroma contained cystic uterine glands in HFD fed mice (arrows) that were dilated relative to those in the ND control. The thickness of endometrium was calculated and indicated increases of uterine thickness in HFD group. The results were reported as mean ± SD (n = 6, * *P* < 0.05). **B** H&E stained ovary sections. There were differentially developing follicles including primordial follicles (Yellow arrow indicated), growing follicles (Blue arrow indicated), antral follicles (Black arrow indicated) and atretic follicles/dilated follicular vacuoles (asterisks indicated) in the ovary sections and the numbers of the follicles in different periods were calculated. Quantitative analysis results were presented as mean ± SD (n = 6, *P < 0.05). **C** H&E stained oviduct sections. The HFD fed mice oviduct structure had a thickened walls and loosely arranged stromal cells in comparison to their ND fed counterpart. The thickness of oviduct was presented as mean ± SD (n = 6, *P < 0.05). Scale bar = 100 μm, 200 μm, respectively

Morphological analyses of the H&E stained uterine sections of the HFD group showed extensive hyperplasia. Most notable the cystic uterine glands were dilated, and the stromal layer was thickened (Fig. 4A). Moreover, the epithelial cells lining the endometrium lost their normal columnar shape and the nuclei appeared round (arrows indicated in Fig. 4A), which is reflective of atypical hyperplasia. The ovarian sections of the HFD fed mice displayed abnormal follicular development (arrows indicated in Fig. 4B) and numerous vacuoles (asterisks indicated in Fig. 4B) which were dispersed throughout all of the ovarian sections. Meanwhile, the number of follicles in different periods were calculated and the results showed a slight increase of atretic follicles in HFD group (Fig. 4B), indicating potential impairment in the follicular development. Additionally, in HFD group, the oviduct walls were thickened and the mucosal stromal layer appeared more loosely arranged (arrow sizes in indicated in Fig. 4C) compared to counterparts in the ND group (Fig. 4C).

### The expression of pro-inflammatory cytokines in female reproductive organs

**Fig. 5:**
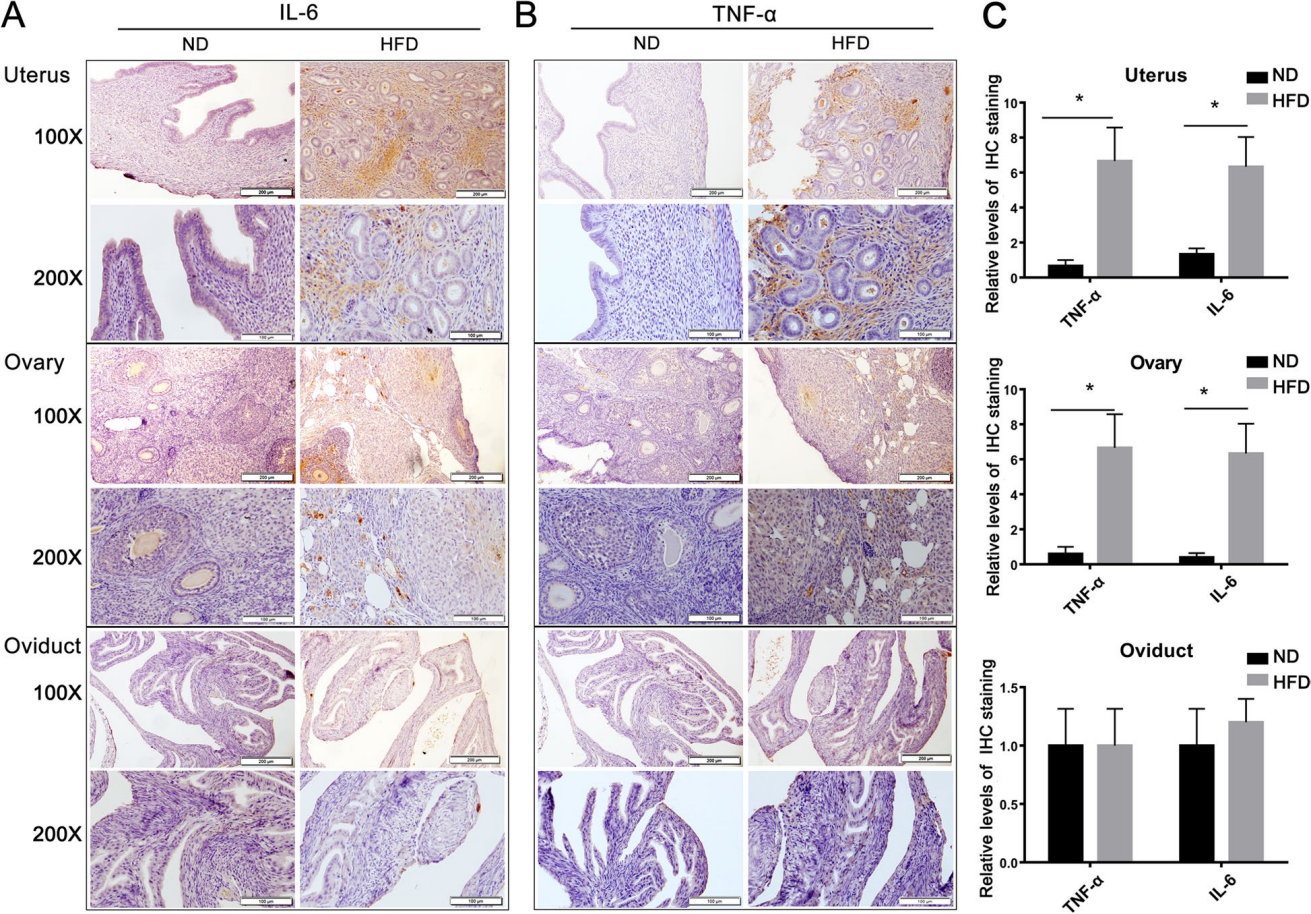
Obesity induced increases in IL-6 and TNF-α levels in reproductive system organs. **A**-**B** IL-6 (**A**) and TNF-α (**B**) immunostaining granules in the stromal layer in the uterus and ovary from HFD fed mice were more intense suggesting higher levels of IL-6 and TNF-α than in the ND fed mice. However, the staining intensity in the oviducts was the same in both the HFD and ND fed groups. Scale bar = 100 μm, 200 μm, respectively. **C** Represents the quantitative analysis of relative expression of IL-6 and TNF-α in tissues. Quantitative analysis results were expressed as mean ± SD (*n* = 5, *P < 0.05)

The impact of diet difference on inflammatory status in the uterus, ovary and oviduct was evaluated by the expression levels of IL-6 and TNF-α. IHC staining of IL-6 and TNF-α in the stromal layers of both the uterus (Fig.5A) and ovary (Fig.5B) in the HFD group was more intense than in the ND group at their 14-week-old. However, the levels of the both pro-inflammatory cytokines in two groups were not different from one another in the oviducts (Fig. 5C).

**Fig. 6:**
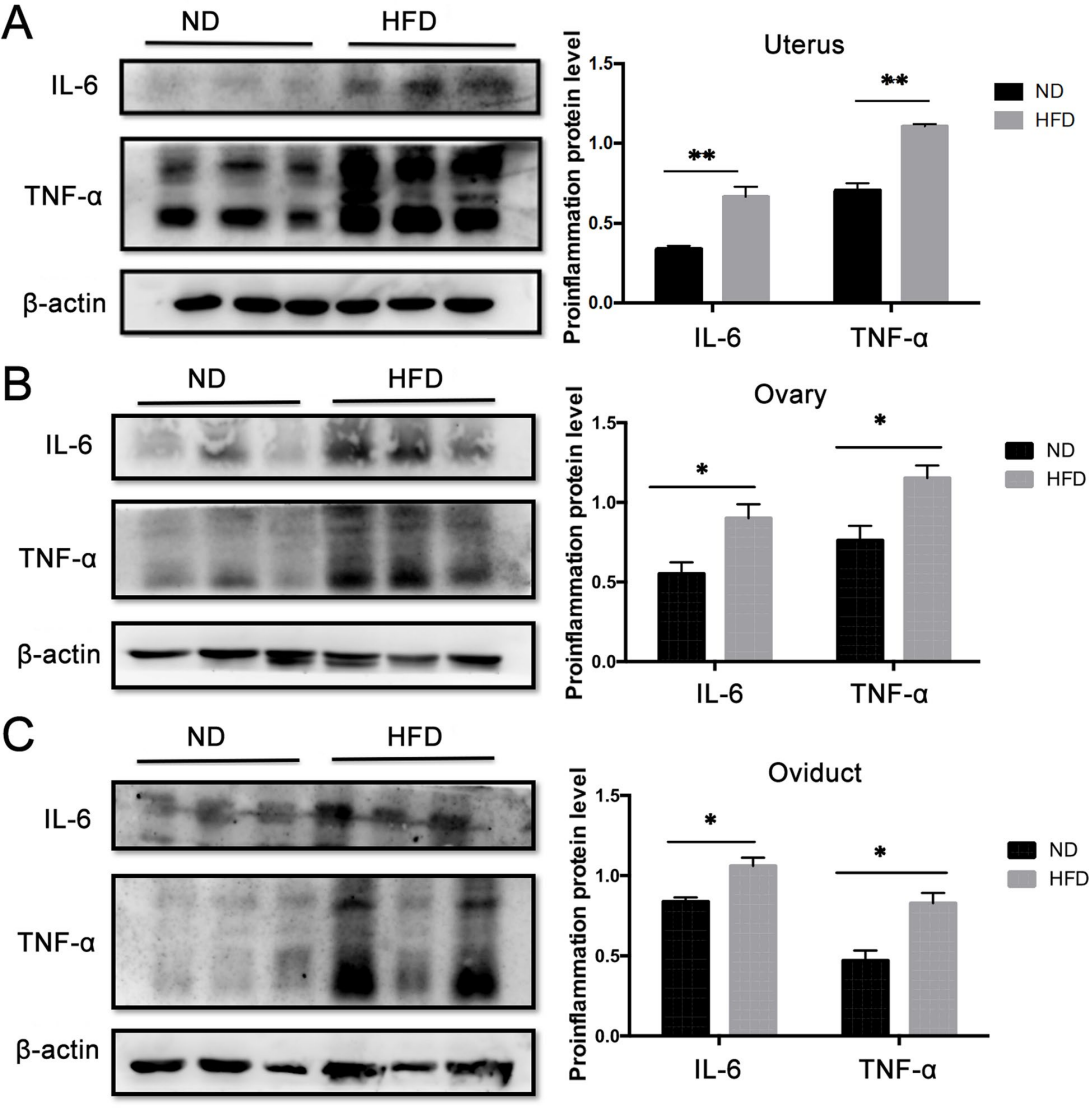
Obesity induced increases in IL-6 and TNF-α protein expression levels in female reproductive system. Comparison of IL-6 and TNF-α protein expression levels by Western blot analysis in uterus (**A**), ovary (**B**) and oviduct (**C**) in HFD and ND fed mice. IL-6 and TNF-α protein expression levels were dramatically greater in the tissues of the HFD group than the ND group (n = 6). Data were normalized to β-Actin expression levels validated loading level equivalence and presented as mean ± SD (n = 6). *P < 0.05, **P < 0.01

Meanwhile, Western blot analysis of IL-6 and TNF-α in the uterus (Fig. 6A), ovary (Fig. 6B) and oviduct (Fig. 6C) clearly showed that their expression levels were markedly higher in the HFD group than in the ND group. Such changes are in agreement with the results of IHC analysis.

**Fig. 7:**
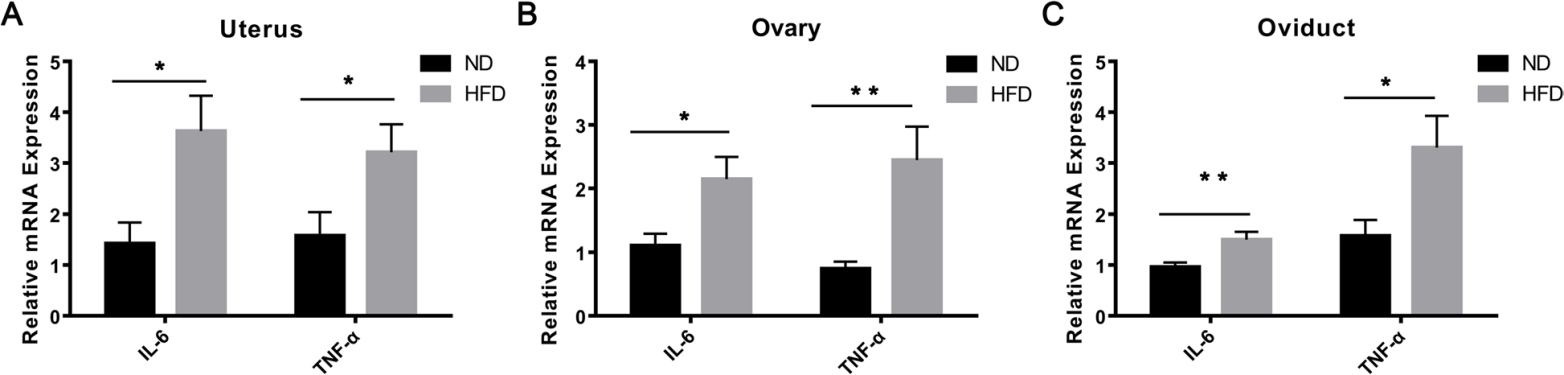
Increase of IL-6 and TNF-α mRNA expression levels in reproductive tissues of obese mice*.* IL-6 and TNF-α mRNA levels evaluated by RT-qPCR in uterus (**A**), ovary (**B**) and oviduct (**C**) of HFD and ND diet fed mice. Higher levels of these two pro-inflammatory cytokines are evident in reproductive tissues from HFD fed mice than those in the ND control fed group. Data were presented as mean ± SD (*n* = 10). *P < 0.05, **P < 0.01

Additionally, qRT-PCR results showed that IL-6 and TNF-α mRNA levels were significantly increased in the HFD fed group reproductive tissues relative to those in normal controls. In the uterus, the IL-6 and TNF-α mRNA expression levels in the HFD mice were 3.63 ± 2.21 and 3.22 ± 1.73, respectively, whereas they were 1.43 ± 1.31 and 1.58 ± 1.43 in the ND fed counterparts (*n* = 10, *P < 0.05; Fig. 7A). In the ovary, the IL-6 and TNF-α mRNA expressions in the HFD mice were 2.15 ± 1.11 and 2.45 ± 1.66, respectively, whereas they were 1.11 ± 0.6 and 0.75 ± 0.34 in the ND fed counterparts (n = 10, *P < 0.05; Fig. 7B). In the oviduct, the IL-6 and TNF-α mRNA expressions were 1.50 ± 0.47 and 3.31 ± 1.88 in HFD mice, respectively, whereas they were 0.96 ± 0.27 and 1.58 ± 0.99 in the ND fed counterparts (n = 10, *P < 0.05, **P < 0.01; Fig. 7C).

These elevated pro-inflammatory cytokine mRNA and protein expression levels are indicative of the development of chronic inflammatory status in mice fed with HFD. This change likely contributes to the pathological changes expressed in their female reproductive organs.

### Obesity effects on JNK, ERK1/2, p38 signaling pathways, NF-κB expressions and phosphorylation status in reproductive tissues

**Fig. 8:**
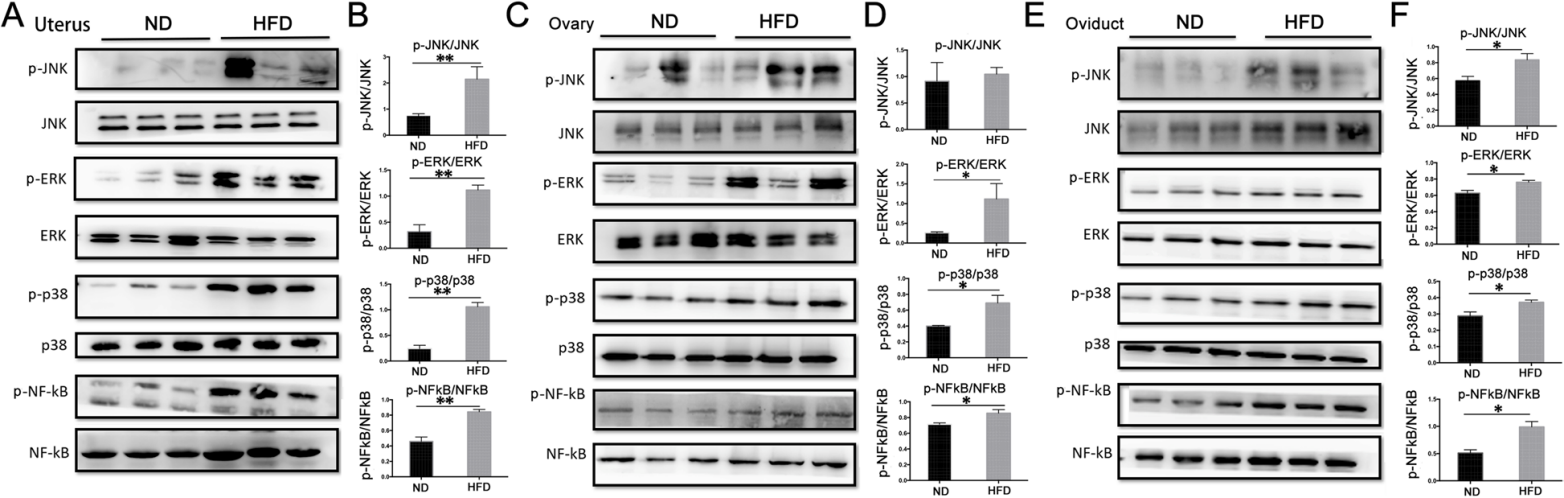
Alterations of inflammatory status in female reproductive tissues linked MAPK and NF-κB signaling pathways. MAPKs and NF-κB signaling pathway activation was monitored based on changes in JNK, ERK1/2, p38 and NF-κB protein expression levels in conjunction with changes in their phosphorylation status. Western blot analyses indicated marked increases in protein expressions of both MAPK subfamily constituents and NF-κB downstream in the uterus (**A** and **B**) and oviduct (**E** and **F**, n = 6). Similarly corresponding rises also occurred in their phosphorylation levels in the uterine and oviduct tissues (**A**, **B**, **E** and **F**, n = 6) from obese mice in comparison to their ND counterpart. Meanwhile, the ERK1/2, p38, NF-κB levels and their phosphorylation status (p-ERK1/2, p-p38 and p-NF-κB) also dramatically increased in the ovary of the mice on a HFD mice, whereas no significant changes occurred in neither the JNK expression level nor its phosphorylation status (**C** and **D**, n = 6). Data were presented as mean ± SD (n = 6), *P < 0.05, **P < 0.01

Representative pro-inflammatory cytokine IL-6 and TNF-α mRNA and protein levels rose in obese reproductive tissues. One possibility is that such changes result from or in MAPK and NF-κB signaling pathway activation. To confirm signaling pathway activation, representative constituents of the three different branches of the MAPK super family and the downstream NF-κB expression levels and phosphorylation status were compared in the two groups. The JNK, ERK1/2, p38, NF-κB expression levels and their phosphorylation status (p-JNK, p-ERK1/2, p-p38 and p-NF-κB) markedly increased in the uterine and oviduct tissues of mice on a HFD (Fig. 8A, B, E, F). Meanwhile, the ERK1/2, p38, NF-κB levels and their phosphorylation status (p-ERK, p-p38 and p-NF-κB) also dramatically increased in the ovary of the mice on a HFD, whereas no significant changes occurred in neither the JNK expression level nor its phosphorylation status (Fig. 8C, D).

## Discussion

Obesity is a multifactorial disease whose contributing factors include genetic predisposition and ingestion of foods having a high fat content. Such excesses combined with insufficient physical activity promote development of obesity while the genetic influence affects individual susceptibility to dietary intake [31]. Typical characteristics of obesity include excessive body mass and excessive intake of fat laden nutrients that are stored rather than metabolized for energetic needs.

In humans, the definition of a HFD includes plans in which 30 to 70% of their caloric value is derived from fats [32]. In order to simulate the conditions leading to human obesity, we generated the female obese HFD mouse model by providing a diet in which fats provided 60% of the caloric value whereas in the ND control group it was reduced to 10%. The results showed that both the body weight and abdominal fat mass increased in the HFD fed mice relative to those fed on a ND. These results are somewhat consistent with a previous report showing that adipocyte volume expansion and their abundance can both increase as a consequence of excessive food consumption [33]. Such rises occur because adipocytes function as fat depots [33]. In this study, we also verified that the HFD is a relevant obesity model because it led to fat store accumulation in the female reproductive system. This observation are in agreement with increases in serum lipid profiles involving rises in total CHOL, HDL and LDL. On the other hand, the liver is the major organ for lipid metabolism and lipid accumulation reaches excessive levels on a HFD. These increases can definitely damage its morphological structure and function, finally leading to many hepatic diseases such as fatty liver and hepatocirrhosis. Moreover, many fat vacuoles appeared in the hepatocytes and a severe hepatic steatosis developed in mice fed the HFD indicating establishment of a relevant HFD model.

Recently, it has become apparent that there are many life threatening diseases associated with obesity, including diabetes mellitus, cardiovascular diseases, cancers, hepatic dysfunction, etc. [2]. In general, female obese patients frequently suffer from several kinds of gynecological disorders such as endocrine dyscrasia, polycystic ovary syndrome (PCOS), amenorrhoea and even infertility [2, 3, 6]. Our results of breeding trials showed that the number of pups delivered by obese mice was significantly reduced in the HFD fed group relative to those on the ND. Due to the known detrimental effects of obesity on female fertility, we characterized its harmful effects on the female reproductive organ morphology. In the HFD group, huge fat droplets accumulated in the stromal layers of the uterus, ovary and oviducts and their histological integrity was extensively altered. The changes included hyperproliferative uterus, vacuolated ovarian tissue and thickened oviduct walls.

In our study, obesity-induced damage to the female reproductive system is associated with increases in IL-6 and TNF-α mRNA and protein expression levels based on an agreement in the results of IHC staining, Western blotting and quantitative RT-PCR in the HFD female reproductive organs. Such increases were relevant to MAPK signaling activation and increases in NF-κB expression. Many previous studies reported that adipose tissue acts as a dynamic endocrine organ, which can secrete numerous pro-inflammatory cytokines such as IL-6 and TNF-α [4]. On the other hand, obesity was recently recognized as a low-grade chronic inflammatory status [33], and IL-6 and TNF-α are considered as biomarkers of this condition. These cytokines have pivotal roles in mediating inflammation, hematopoiesis, cell proliferation and apoptosis [34–36]. Accordingly, our results indicate that mice fed a HFD acquire a chronic inflammatory status during obesity development. This outcome shows that the HFD model is relevant to delineating how infertility develops in the human obese population [12, 25, 37, 38], because in obese human females fat accumulates in adipocytes and other organs or tissues such as liver, smooth and skeletal muscles [39].

The ERK1/2, p38 and JNK signaling pathway control can become maladaptive inducing responses associated with a wide range of diseases including cancers, ischemic heart disease, autoimmune diseases, etc. [40]. Different cytokines or growth factors interacting with their cognate receptors mediate control of cell proliferation and differentiation, inflammation through modulating either cell cycle progression or transcription factors suppressing tumor formation [41, 42]. In females, this activated pathway induced by inositol can lead to endothelial dysfunction in preeclampsia [43] and it also plays a critical role in the pathogenesis of PCOS, but it decreases the steroidogenic response to gonadotropins in preovulatory granulosa cells [44]. On the other hand, ERK1/2 inhibition along with activation of either p38 and/or JNK can lead to increases in inflammation and atherosclerosis.

p38 and JNK can be activated by various stress stimuli such as UV, which induce apoptosis [45]. p38 is an oxidative stress-response kinase and its role in the female reproductive organs is quite complex during the entire pregnancy process and parturition. p38 MAPK is relevant to some processes such as decidualization, trophoblast differentiation and invasion [46], myometrial quiescence or activation during parturition, and placental growth [47]. Pathologic activation of the p38 pathway can cause adverse pregnancy outcomes including preterm birth [48]. Maladaptive JNK signal pathway activation is also associated with numerous female reproductive diseases. For example, solely inducing sustained JNK/AP-1 signaling pathway activation is sufficient to induce delivery, however, LPS-induced rises in TNF-α expression levels lead to inappropriate JNK pathway activation, which usually results in premature delivery [49]. Besides, activated JNK induced by oxidative stress is also associated with granulosa cell apoptosis [50].

It is known that maladaptive MAPKs signaling pathway activation induces adipogenesis. However, the involvement of the role of the ERK1/2 signaling pathway in this process is somewhat controversial. Some studies claimed that this pathway instead inhibits adipogenesis [51], whereas others suggested that it promotes adipogenesis [52]. Moreover, some reports indicated that this pathway promotes adipogenesis in the initial stage whereas in the later stages it has a negative role in this process [53]. Additionally, a JNK1 deficient mouse is reported to contribute to obesity development suggesting lack of JNK1 involvement in this process [54, 55] and p38 signaling pathway activation can enhance the adipogenesis [56, 57]. Taken together, there is substantive evidence that the MAPK signaling pathway activation by various stressors contributes to inducing obesity by enhancing adipogenesis.

NF-kB is a transcription factor mediating signaling pathway control of a myriad responses in health and disease. This transcription factor undergoes activation by hyperlipidemia in obese patients. It regulates the expression of immediate-early response genes involved in stress and inflammation and contributes to various female reproductive diseases [58]. NF-kB activation normally occurs in the myometrium prior to delivery and untimely activated NF-kB leads to premature delivery [59]. More importantly, some studies elucidated crosstalk of NF-kB with other pathways in the pathogenesis of ovarian cancer [60].

Several alternative mechanisms could account for how the HFD induced increases in MAPK signaling and upregulated NF-κB expression which led to adipocyte hypertrophy and hyperplasia in the female reproductive system. a) Direct activation by IL-6 and TNFα of MAPK signaling and upregulation of NF-κB expression. IL-6 could induce such responses via directly activating many signaling pathways such as NF-kB, MAPKs, PI3K, mTOR and AMPK [36]. b) Direct activation by IL-6 and TNFα of their cognate receptors, which in turn increases MAPK signaling and upregulates NF-κB expression. For example, rises in TNF-α gene and protein expression levels stemming from the altered lipid profile in pathological adipogenesis could induce increases in MAPK signaling and upregulate NF-κB expression through interacting with its cognate receptors, TNFR 1 or TNFR2 [61]. c) One or more of the constituents in the altered lipid profile may stimulate non-cognate receptors and subsequently increase MAPK signaling and NF-κB expression. All three of these possibilities are tenable since in different tissues one or more of these three alternatives accounts for how adipogenesis increases IL-6 and TNFα expression levels.

**Fig. 9:**
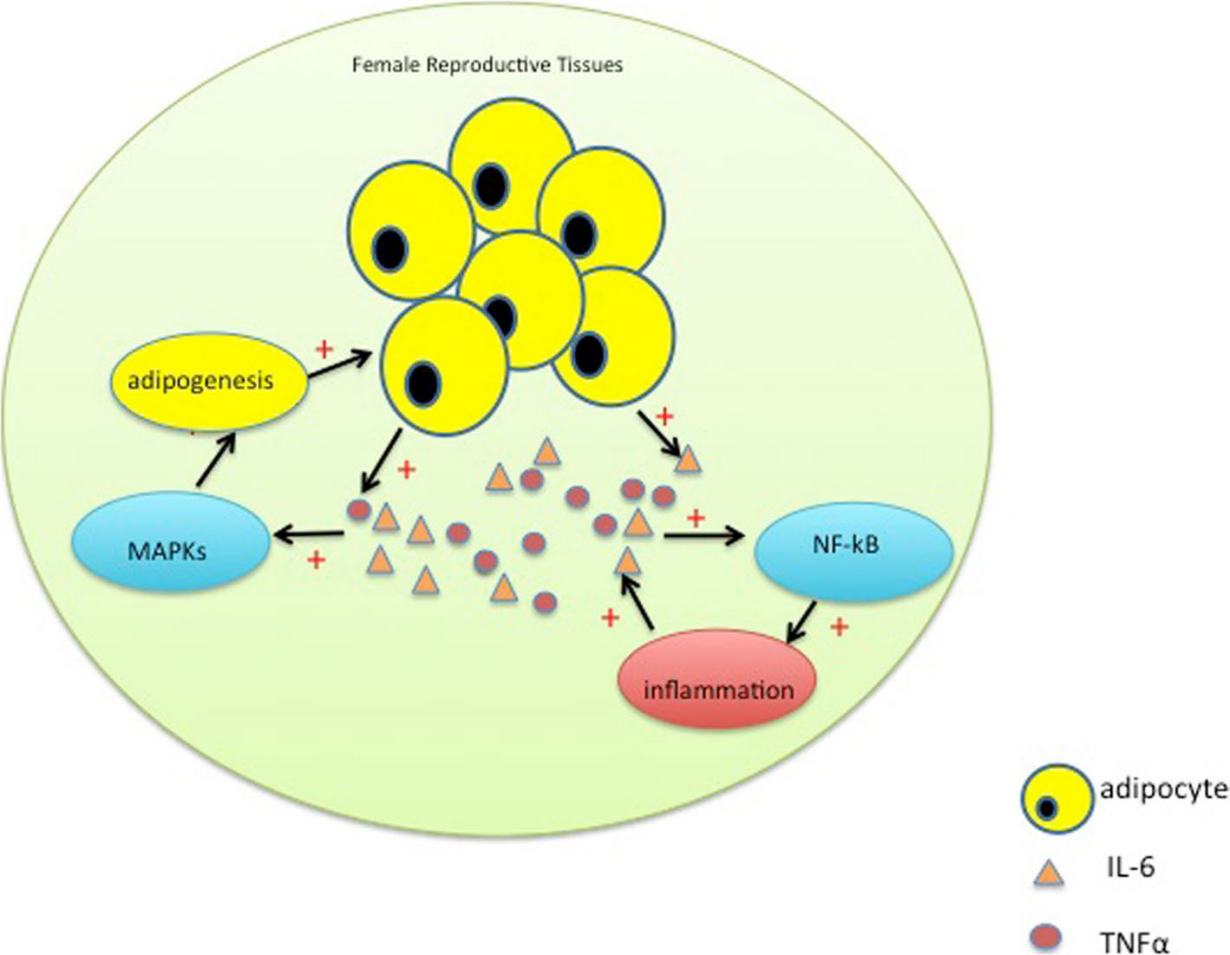
Schematic model showing the signaling pathways mediating structural and functional changes in the female reproductive system in obese mice. In obese females, their reproductive system became lipid droplet laden and secretes excessive pro-inflammatory cytokines such as IL-6 and TNF-α. These heightened secretions damage the morphological structures and directly disrupt normal biological functions of the reproductive organs. It is presumed that either IL-6 or TNF-α directly induce increases in JNK, ERK/12 and p38 signaling and NF-kB stem or these pro-inflammatory cytokines induce these effects through cognate receptor activation. In any case, these pro-inflammatory cytokines can activate NF-kB-linked signaling pathway, which aggravates the adverse inflammatory status, whereas activation of the MAPKs signaling pathway augments adipogenesis, which finally leads to hypertrophy and hyperplasia of adipocytes in the female reproductive system

Herein, it is evident that the increases in fat droplets are associated with rises in the IL-6 and TNF-α expression levels and damage to the reproductive organ structural features and functions of the fat laden uterus, ovary and oviduct. Any or all of these effects can contribute to reducing fertility and reproductive success. Furthermore, the activated NF-kB pathway is involved in inducing immune responses that can aggravate increases in the inflammatory status in the uterus, ovary and oviduct, whereas activation of the MAPKs signaling pathway augments adipogenesis in the female reproductive system (Fig. 9).

## Conclusions

HFD induced obesity contributes to the development of an inflammatory status in female reproductive system. Such an effect is associated with both morphological impairment and functional defects in the uterus, ovary and oviduct, leading to female subfertility or infertility. These findings suggest that designing therapeutic strategies to reduce the inflammatory status in obese patients may provide an option for reducing subfertility and infertility in a clinical setting.

## Supplementary Information

**Additional file 1: Supplementary Table 1.** The ingredient of high-fat diet and normal diet.

## Data Availability

All data generated or analyzed during this study are included in this published article and its supplementary information files.

## Abbreviations

CHOL: cholesterol
FSH: follicle-stimulating hormone
HDL: high density lipoprotein
H&E: hematoxylin and eosin
HFD: high fat diet
IHC: immunohistochemistry
IL-6: interleukin-6
LDL: low density lipoprotein
LH: luteinizing hormone
MAPK: mitogen-activated protein kinase
ND: normal diet
NF-kB: nuclear factor kappa B
PCOS: polycystic ovary syndrome
SD: standard deviation
TGL: triglycerides
TNF-α: tumor necrosis factor-alpha
